# (Phenyl­iodos­yl)benzene tosyl­ate dihydrate

**DOI:** 10.1107/S2414314622005223

**Published:** 2022-05-20

**Authors:** Timothy J. Smith, Gerald Koser, Yi Chen, Matthias Zeller, Rocco Iacino, Nichole Selzer

**Affiliations:** a School of Math and Science, Walsh University, 2020 East Maple Street, North Canton, OH 44720, USA; b Department of Chemistry, University of Akron, 190 E. Buchtel Ave, Akron, OH 44304, USA; c Department of Chemistry, Purdue University, 560 Oval Dr., West Lafayette, IN 47907-2084, USA; Benemérita Universidad Autónoma de Puebla, México

**Keywords:** crystal structure, λ^5^-iodinane, di­aryl­iodosyl salt, sulfide oxidation

## Abstract

The structure of the title salt (systematic name: oxodiphenyl-λ^5^-iodanylium 4-methyl­benzene­sulfonate dihydrate), C_12_H_10_IO^+^·C_7_H_7_O_3_S^−^·2H_2_O, at 150 K, has monoclinic (*P*2_1_/*c*) symmetry. The mol­ecular structure features an angular (phenyl­iodos­yl)benzene cation, the geometry of which was hitherto undescribed in the literature: in the cation, both I—C bonds are approximately normal to the I=O bond, forming a C—I—C angle of 95.36 (4)°. The crystal structure displays O—H⋯O, O—H⋯I and O—H⋯S hydrogen bonding.

## Abstract 2

The title compound is a λ^5^-di­aryl­iodosyl salt capable of sulfide oxidations.

## Graphical abstract

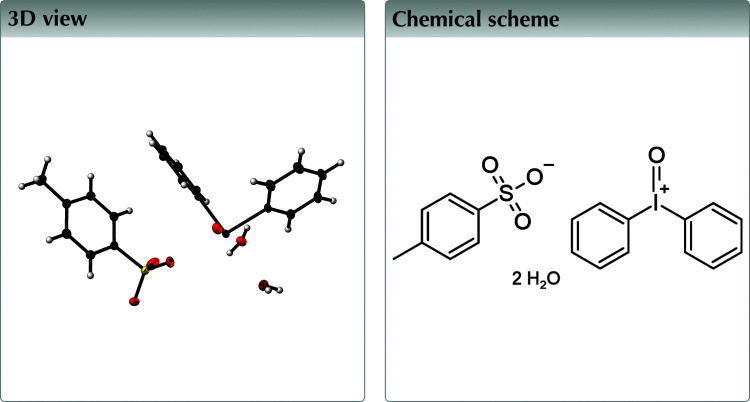

## Structure description

**Figure 1:**
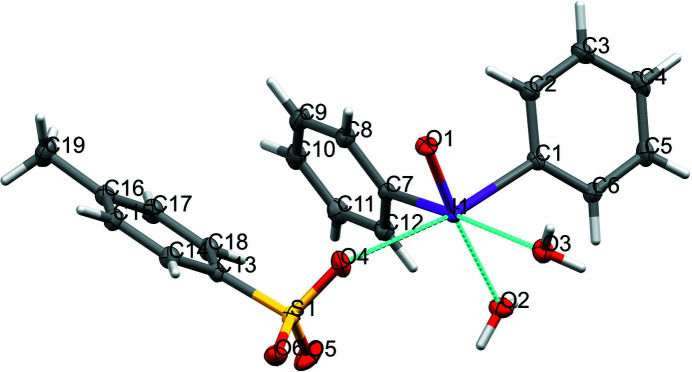
The title compound **1** with 50% displacement ellipsoids. All hydrogen labels are omitted for clarity.

**Figure 2:**
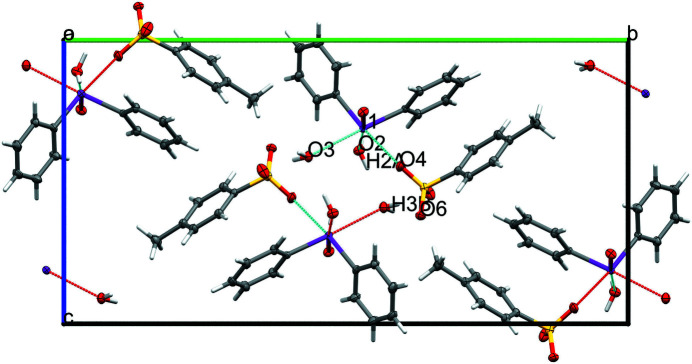
The unit cell as viewed down the *a* axis showing inter­molecular hydrogen bonds as dashed lines.

**Figure 3:**
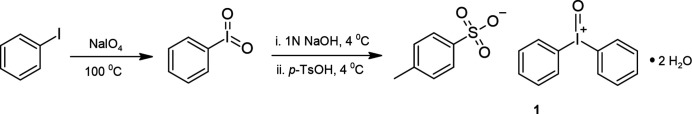
The synthetic route to obtain the title compound **1**.

The crystal structure of (phenyl­iodos­yl)benzene tosyl­ate dihydrate (**1**) is shown in Fig. 1 ▸. A partial packing structure is shown in Fig. 2 ▸. The title compound **1** crystallizes from water *via* slow cooling to 293 K to the monoclinic crystal system with space group *P*2_1_/c. This iodosyl salt arose from the reaction of iodoxybenzene with sodium hydroxide (Fig. 3 ▸). The inter­mediate formed was captured with *p*-toluene sulfonic acid, generating **1**.

In compound **1** there are two phenyl rings connected to the iodine centre, C1—I1— C7, with a bond angle of 95.36 (4)°. The bond lengths of C1—I1 and C7—I1 are 2.1289 (11) and 2.1370 (12) Å, respectively. These values are comparable to the sum of the van der Waals radii (2.05 Å; Bondi, 1964 ▸). These bond lengths are comparable to those found in 1,1,1-tri­acetoxy1,1-di­hydro-1,2-benzo­iodoxol-3(1*H*)-one, *i.e*. the Dess–Martin periodinane, with a C—I bond length between the phenyl and iodine of 2.1025 (16) Å (Schröckeneder *et al.*, 2012 ▸). A secondary bonding inter­action with the *p*-toluene sulfonate anion, O4⋯I1 [2.7076 (10) Å], resides nearly perpendicular at 77.55 (4)° to the I1—O1 bond. This bond length is shorter than the sum (3.05 Å) of the covalent radii, and this is analogous to secondary bonding that was observed by Rentzeperis in his bis­(di­phenyl­iodo­nium *I*-oxide) di­acetate trihydrate, between the acetate anion and the iodo­nium *I*-oxide centre (Bozopoulos & Rentzeperis, 1987 ▸). Zhdankin found similar bond lengths of coordination between the tri­fluoro­methane­sulfonate oxygen anion and the cationic iodo­nium centre (2.797 Å) in [(aryl­sulfon­yl)meth­yl](phen­yl)iodo­nium tri­fluoro­methane­sulfonate (Zhdankin *et al.*, 1997 ▸). Additionally, Rentzeperis found similar coordinating distances with this acetate ion [2.449 (7) Å] and the oxygen atoms of the three water mol­ecules, at 2.449 (7), 2.732 (9) and 2.732 (7) Å, respectively (Bozopoulos & Rentzeperis, 1987 ▸). In the title compound, the I1—O1 bond of 1.8108 (9) Å indicates double-bond character, as the computed double-bond length *via* van der Waals radii predicts 1.86 Å (Bondi, 1964 ▸). Rentzeperis found a similar bond length, 1.842 (6) Å, in bis­(di­phenyl­iodo­nium *I*-oxide) di­acetate trihydrate (Bozopoulos & Rentzeperis, 1987 ▸).

Zhdankin synthesized a tosyl­ate derivative of 2-iodoxybenzoic acid. Crystals of the final product could not be isolated, but the inter­mediate mixed tosyl­ate-acetate derivative was analysed. The I—O bond lengths in this iodine(V) compound had distances of 2.080 (2), 2.213 (2), 2.027 (2) and 1.998 (2) Å (Yusubov *et al.*, 2013 ▸). These values are in accordance with a single-bond inter­action of iodine with oxygen, further indicating that the I—O bond distance in **1** is of a double-bond nature. Additionally, bond lengths being shorter than predicted in hypervalent iodine compounds have been studied previously (Koser *et al.*, 1976 ▸). Koser confirmed the I—O single bond length was shorter (1.91 Å) than the computed distance (1.96 Å) in his seminal work on hy­droxy(tos­yloxy)iodo­benzene. Additionally, in **1**, secondary coordination of the iodo­nium *I*-oxide centre with neighbouring water mol­ecules indicates a close contact *via* the I1⋯O2 and I1⋯O3 with bond distances of 2.5674 (10) and 2.8118 (10) Å, respectively.

The title compound forms a distorted octa­hedral geometry in accordance with comparison to a VSEPR model. The O1—I1—O2 bond angle of 175.27 (4)°, the C7—I1—O3 angle of 176.33 (4)°, the O1—I1—O4 angle of 77.55 (4)° with the coordinating tosyl­ate anion and the C1—I1—C7 angle of 95.36 (4)° complete the distorted octa­hedral geometry. The accompanying tosyl­ate anion and water mol­ecules occupy apical and equatorial positions to stabilize the monomeric complex. Bis(di­phenyl­iodo­nium *I*-oxide) di­acetate trihydrate also adopted a distorted octa­hedral geometry, albeit *via* a dimeric coordinating structure (Bozopoulos & Rentzeperis, 1987 ▸). In this complex, the asymmetric units form distorted trigonal–pyramidal arrangements, where the iodine atoms occupy the apices, resembling the IO_3_^−^ iodate anion. Secondary I⋯O inter­actions complete the distorted octa­hedral geometry around each individual iodine atom. The title complex **1** does not dimerize like the Rentzeperis compound, most likely due to the bulky nature of the coordinating tosyl­ate anion, along with additional hydrogen bonding of the sulfone O atoms and water O atoms with neighbouring water mol­ecules.

Examination of the mol­ecular packing as illustrated in Fig. 2 ▸ shows O3^i^⋯H2*A* and O6^iii^⋯H3*B* contacts, with O⋯H distances of 1.92 (2) and 1.99 (2) Å, respectively, as viewed down the *a* axis (Fig. 2 ▸ and Table 1 ▸). These two coordinations inhibit aggregation of the iodo­nium centres as seen in bis­(di­phenyl­iodo­nium *I*-oxide) di­acetate trihydrate (Bozopoulos & Rentzeperis, 1987 ▸).

## Synthesis and crystallization

(Phenyl­iodos­yl)benzene tosyl­ate dihydrate was synthesized according to a modified procedure by Chen (2007 ▸) and is illustrated in Fig. 3 ▸. Iodo­benzene (2.04 g, 10 mmol) was added to a water solution (20 ml) of sodium metaperiodate (4.7 g, 22 mmol) with a small amount of toluene (0.3 ml) to minimize steam distillation. The reaction was heated to reflux for 18 h and then cooled to room temperature. To the cooled reaction flask were added 50 ml of ice-cold water, and the white crystals that formed were filtered, washed with cold water (20 ml), cold chloro­form (10 ml), and air-dried in a dark room until a constant weight was found (2.08 g, 8.81 mmol, 88% yield). The crude material was used in the next step without further purification. The iodoxybenzene (2.08 g, 8.81 mmol) was added to a stirred solution of 1 N NaOH (18.7 ml) pre-cooled to 277 K. The reaction was stirred for 1 h maintaining the temperature of the reaction below 281 K. The NaIO_3_ that formed was filtered off. The filtrate was poured into a round-bottomed flask equipped with a magnetic stir bar and cooled to 277 K. With vigorous stirring *p*-toluene sulfonic acid monohydrate (2.87 g, 15.08 mmol) was added to the cooled filtrate and a white precipitate formed. The suspension was stirred for an additional 30 min and then filtered. The compound was washed with a minimal amount of diethyl ether (10 ml) and ice-cold water (10 ml). The product (1.75 g, 3.47 mmol, 47.5% yield) matched known ^1^H, ^13^C and FTIR data (Chen, 2007 ▸). A sample for crystallographic analysis was prepared by dissolving the sample in a minimal amount of boiling water and allowing for slow cooling in an insulated thermal bath, insuring that the temperature took at least two days to return to room temperature. After additional cooling at room temperature for four days, the crystals that formed were suitable for X-ray analysis.

## Refinement

Crystal data, data collection and structure refinement details are summarized in Table 2 ▸.

## Supplementary Material

Crystal structure: contains datablock(s) I, global. DOI: 10.1107/S2414314622005223/bh4069sup1.cif

Structure factors: contains datablock(s) I. DOI: 10.1107/S2414314622005223/bh4069Isup3.hkl

Supporting information file. DOI: 10.1107/S2414314622005223/bh4069Isup3.cml

CCDC reference: 2173122

Additional supporting information:  crystallographic information; 3D view; checkCIF report

## Figures and Tables

**Table 1:** Hydrogen-bond geometry (Å, °)

*D*—H⋯*A*	*D*—H	H⋯*A*	*D*⋯*A*	*D*—H⋯*A*
O2—H2*A*⋯O3^i^	0.86 (2)	1.92 (2)	2.7563 (14)	165 (2)
O2—H2*B*⋯I1^ii^	0.85 (2)	3.14 (2)	3.9397 (10)	158.1 (18)
O2—H2*B*⋯O1^ii^	0.85 (2)	1.91 (2)	2.7443 (14)	169 (2)
O3—H3*A*⋯S1^i^	0.84 (2)	3.00 (2)	3.7847 (11)	155.7 (17)
O3—H3*A*⋯O5^i^	0.84 (2)	1.92 (2)	2.7625 (15)	174.6 (19)
O3—H3*B*⋯S1^iii^	0.79 (2)	2.89 (2)	3.5812 (10)	147.5 (18)
O3—H3*B*⋯O6^iii^	0.79 (2)	1.99 (2)	2.7776 (14)	171 (2)

Symmetry codes: (i) 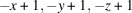 ; (ii) 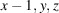 ; (iii) 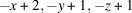 .

**Table 2:** Experimental details

Crystal data	
Chemical formula	C_12_H_10_IO^+^·C_7_H_7_O_3_S^−^·2H_2_O
*M*_r_	504.32
Crystal system, space group	Monoclinic, *P*2_1_/*c*
Temperature (K)	150
*a*, *b*, *c* (Å)	6.1823 (3), 24.9509 (11), 12.7606 (6)
β (°)	100.257 (2)
*V* (Å^3^)	1936.92 (16)
*Z*	4
Radiation type	Mo *K*α
μ (mm^−1^)	1.79
Crystal size (mm)	0.23 × 0.18 × 0.15

Data collection	
Diffractometer	Bruker AXS D8 Quest diffractometer with PhotonII charge-integrating pixel array detector (CPAD)
Absorption correction	Multi-scan (*SADABS*; Bruker, 2021 ▸)
*T*_min_, *T*_max_	0.644, 0.747
No. of measured, independent and observed [*I* > 2σ(*I*)] reflections	55248, 7429, 6815
*R*_int_	0.033
(sin θ/λ)_max_ (Å^−1^)	0.772

Refinement	
*R*[*F* ^2^ > 2σ(*F* ^2^)], *wR*(*F* ^2^), *S*	0.018, 0.042, 1.09
No. of reflections	7429
No. of parameters	258
H-atom treatment	H atoms treated by a mixture of independent and constrained refinement
Δρ_max_, Δρ_min_ (e Å^−3^)	0.49, −0.39

Computer programs: *APEX4* and *SAINT* (Bruker, 2021 ▸), *SHELXT2018/2* (Sheldrick, 2015*a* ▸), *SHELXL2018/3* (Sheldrick, 2015*b* ▸), *Mercury* (Macrae *et al.*, 2020 ▸) and *publCIF* (Westrip, 2010 ▸).

## full crystallographic data

### Crystal data

**Table:**

C_12_H_10_IO^+^·C_7_H_7_O_3_S^−^·2H_2_O	*F*(000) = 1008
*M**_r_* = 504.32	*D*_x_ = 1.729 Mg m^−^^3^
Monoclinic, *P*2_1_/*c*	Mo *K*α radiation, λ = 0.71073 Å
*a* = 6.1823 (3) Å	Cell parameters from 9659 reflections
*b* = 24.9509 (11) Å	θ = 3.4–33.2°
*c* = 12.7606 (6) Å	µ = 1.79 mm^−^^1^
β = 100.257 (2)°	*T* = 150 K
*V* = 1936.92 (16) Å^3^	Fragment, colourless
*Z* = 4	0.23 × 0.18 × 0.15 mm

### Data collection

**Table:**

Bruker AXS D8 Quest diffractometer with PhotonII charge-integrating pixel array detector (CPAD)	7429 independent reflections
Radiation source: fine focus sealed tube X-ray source	6815 reflections with *I* > 2σ(*I*)
Triumph curved graphite crystal monochromator	*R*_int_ = 0.033
Detector resolution: 7.4074 pixels mm^-1^	θ_max_ = 33.3°, θ_min_ = 2.9°
ω and φ scans	*h* = −9→9
Absorption correction: multi-scan (SADABS; Bruker, 2021)	*k* = −38→38
*T*_min_ = 0.644, *T*_max_ = 0.747	*l* = −19→19
55248 measured reflections	

### Refinement

**Table:**

Refinement on *F*^2^	Secondary atom site location: difference Fourier map
Least-squares matrix: full	Hydrogen site location: mixed
*R*[*F*^2^ > 2σ(*F*^2^)] = 0.018	H atoms treated by a mixture of independent and constrained refinement
*wR*(*F*^2^) = 0.042	*w* = 1/[σ^2^(*F*_o_^2^) + (0.0115*P*)^2^ + 1.2666*P*] where *P* = (*F*_o_^2^ + 2*F*_c_^2^)/3
*S* = 1.09	(Δ/σ)_max_ = 0.004
7429 reflections	Δρ_max_ = 0.49 e Å^−^^3^
258 parameters	Δρ_min_ = −0.39 e Å^−^^3^
0 restraints	Extinction correction: SHELXL2018/3 (Sheldrick, 2015b), Fc^*^=kFc[1+0.001xFc^2^λ^3^/sin(2θ)]^-1/4^
Primary atom site location: dual	Extinction coefficient: 0.00186 (16)

### Special details

**Table:**

Refinement. H atoms attached to carbon were positioned geometrically and constrained to ride on their parent atoms. C—H bond distances were constrained to 0.95 Å for aromatic CH moieties, and to 0.98 Å for the CH_3_ group, respectively. Water H atom positions were freely refined. *U*_iso_(H) values were set to a multiple of *U*_eq_(carrier C/O), with 1.5 for CH_3_ and OH, and 1.2 for CH units, respectively.

### Fractional atomic coordinates and isotropic or equivalent isotropic displacement parameters (Å^2^)

**Table:**

	*x*	*y*	*z*	*U*_iso_*/*U*_eq_	
I1	0.68788 (2)	0.46909 (2)	0.68867 (2)	0.01154 (3)	
S1	0.78701 (5)	0.36098 (2)	0.47914 (2)	0.01522 (5)	
O1	0.97764 (14)	0.46950 (4)	0.74614 (8)	0.01780 (17)	
O2	0.27627 (16)	0.47673 (4)	0.61133 (8)	0.01981 (18)	
H2A	0.242 (3)	0.4622 (8)	0.5495 (17)	0.030*	
H2B	0.173 (3)	0.4724 (8)	0.6458 (17)	0.030*	
O3	0.75786 (17)	0.56665 (4)	0.58959 (8)	0.01987 (18)	
H3A	0.665 (3)	0.5916 (8)	0.5781 (16)	0.030*	
H3B	0.869 (4)	0.5829 (8)	0.6024 (16)	0.030*	
O4	0.86090 (18)	0.40372 (4)	0.55568 (8)	0.02297 (19)	
O5	0.55011 (16)	0.35301 (5)	0.46211 (10)	0.0294 (2)	
O6	0.87304 (16)	0.36783 (4)	0.38086 (7)	0.02007 (18)	
C1	0.5945 (2)	0.51811 (5)	0.80971 (9)	0.0142 (2)	
C2	0.7369 (2)	0.51635 (6)	0.90699 (11)	0.0212 (2)	
H2	0.862288	0.493668	0.917364	0.025*	
C3	0.6902 (3)	0.54883 (6)	0.98885 (11)	0.0249 (3)	
H3	0.783620	0.548237	1.056562	0.030*	
C4	0.5077 (3)	0.58201 (6)	0.97162 (11)	0.0236 (3)	
H4	0.477050	0.604223	1.027645	0.028*	
C5	0.3694 (2)	0.58310 (6)	0.87349 (12)	0.0229 (3)	
H5	0.244730	0.606019	0.862871	0.027*	
C6	0.4115 (2)	0.55088 (5)	0.79013 (10)	0.0182 (2)	
H6	0.317967	0.551429	0.722456	0.022*	
C7	0.6142 (2)	0.39479 (5)	0.75784 (9)	0.0139 (2)	
C8	0.7930 (2)	0.37027 (5)	0.82033 (10)	0.0187 (2)	
H8	0.934473	0.386350	0.830739	0.022*	
C9	0.7592 (3)	0.32126 (6)	0.86755 (11)	0.0235 (3)	
H9	0.878788	0.303693	0.911211	0.028*	
C10	0.5521 (3)	0.29802 (5)	0.85114 (11)	0.0231 (3)	
H10	0.530416	0.264622	0.883500	0.028*	
C11	0.3762 (2)	0.32346 (5)	0.78748 (11)	0.0217 (2)	
H11	0.234867	0.307259	0.776581	0.026*	
C12	0.4048 (2)	0.37264 (5)	0.73933 (10)	0.0178 (2)	
H12	0.285534	0.390249	0.695560	0.021*	
C13	0.90551 (19)	0.30148 (5)	0.53950 (9)	0.0143 (2)	
C14	1.1068 (2)	0.28329 (5)	0.51879 (10)	0.0166 (2)	
H14	1.177697	0.301415	0.468824	0.020*	
C15	1.2027 (2)	0.23823 (5)	0.57228 (10)	0.0180 (2)	
H15	1.339293	0.225549	0.557827	0.022*	
C16	1.1023 (2)	0.21125 (5)	0.64677 (10)	0.0180 (2)	
C17	0.8999 (2)	0.23004 (5)	0.66564 (11)	0.0195 (2)	
H17	0.828525	0.211958	0.715483	0.023*	
C18	0.8014 (2)	0.27479 (5)	0.61256 (10)	0.0172 (2)	
H18	0.663586	0.287143	0.626024	0.021*	
C19	1.2112 (3)	0.16377 (6)	0.70690 (13)	0.0283 (3)	
H19A	1.137702	0.155730	0.767074	0.042*	
H19B	1.366266	0.171982	0.733455	0.042*	
H19C	1.200511	0.132671	0.659362	0.042*	

### Atomic displacement parameters (Å^2^)

**Table:**

	*U*^11^	*U*^22^	*U*^33^	*U*^12^	*U*^13^	*U*^23^
I1	0.00981 (3)	0.01211 (4)	0.01291 (4)	−0.00066 (2)	0.00260 (2)	−0.00161 (2)
S1	0.01308 (12)	0.01470 (12)	0.01821 (13)	0.00026 (10)	0.00368 (10)	0.00096 (10)
O1	0.0086 (3)	0.0232 (4)	0.0211 (4)	−0.0009 (3)	0.0013 (3)	−0.0040 (3)
O2	0.0141 (4)	0.0254 (5)	0.0199 (4)	−0.0026 (4)	0.0030 (3)	−0.0025 (4)
O3	0.0179 (4)	0.0171 (4)	0.0250 (5)	−0.0021 (4)	0.0049 (4)	0.0001 (3)
O4	0.0306 (5)	0.0158 (4)	0.0245 (5)	0.0005 (4)	0.0104 (4)	−0.0040 (4)
O5	0.0127 (4)	0.0304 (6)	0.0440 (6)	0.0006 (4)	0.0024 (4)	0.0130 (5)
O6	0.0234 (4)	0.0212 (4)	0.0160 (4)	0.0004 (4)	0.0045 (3)	0.0021 (3)
C1	0.0147 (5)	0.0131 (5)	0.0153 (5)	−0.0004 (4)	0.0040 (4)	−0.0032 (4)
C2	0.0196 (6)	0.0242 (6)	0.0186 (6)	0.0043 (5)	0.0000 (4)	−0.0052 (5)
C3	0.0286 (7)	0.0280 (7)	0.0170 (6)	0.0019 (6)	0.0013 (5)	−0.0069 (5)
C4	0.0305 (7)	0.0209 (6)	0.0216 (6)	0.0003 (5)	0.0108 (5)	−0.0067 (5)
C5	0.0238 (6)	0.0192 (6)	0.0270 (6)	0.0055 (5)	0.0080 (5)	−0.0035 (5)
C6	0.0177 (5)	0.0174 (5)	0.0192 (5)	0.0029 (4)	0.0026 (4)	−0.0014 (4)
C7	0.0162 (5)	0.0127 (5)	0.0132 (5)	0.0003 (4)	0.0036 (4)	−0.0007 (4)
C8	0.0184 (5)	0.0180 (5)	0.0194 (6)	0.0027 (4)	0.0027 (4)	0.0006 (4)
C9	0.0288 (7)	0.0201 (6)	0.0214 (6)	0.0073 (5)	0.0037 (5)	0.0040 (5)
C10	0.0350 (7)	0.0162 (5)	0.0204 (6)	0.0015 (5)	0.0110 (5)	0.0022 (4)
C11	0.0252 (6)	0.0170 (6)	0.0242 (6)	−0.0047 (5)	0.0084 (5)	−0.0009 (5)
C12	0.0174 (5)	0.0161 (5)	0.0199 (5)	−0.0015 (4)	0.0033 (4)	0.0000 (4)
C13	0.0140 (5)	0.0130 (5)	0.0160 (5)	−0.0011 (4)	0.0028 (4)	−0.0010 (4)
C14	0.0152 (5)	0.0169 (5)	0.0187 (5)	−0.0009 (4)	0.0055 (4)	0.0002 (4)
C15	0.0150 (5)	0.0193 (6)	0.0201 (6)	0.0022 (4)	0.0046 (4)	−0.0005 (4)
C16	0.0208 (6)	0.0152 (5)	0.0181 (5)	0.0013 (4)	0.0037 (4)	−0.0003 (4)
C17	0.0225 (6)	0.0170 (5)	0.0208 (6)	−0.0004 (5)	0.0092 (5)	0.0010 (4)
C18	0.0159 (5)	0.0168 (5)	0.0203 (5)	−0.0001 (4)	0.0069 (4)	0.0001 (4)
C19	0.0334 (8)	0.0234 (7)	0.0292 (7)	0.0094 (6)	0.0090 (6)	0.0083 (5)

### Geometric parameters (Å, º)

**Table:**

I1—O1	1.8108 (9)	C7—C8	1.3849 (17)
I1—C1	2.1289 (11)	C7—C12	1.3883 (17)
I1—C7	2.1370 (12)	C8—C9	1.3954 (19)
I1—O2	2.5674 (10)	C8—H8	0.9500
I1—O4	2.7076 (10)	C9—C10	1.387 (2)
I1—O3	2.8118 (10)	C9—H9	0.9500
S1—O5	1.4555 (10)	C10—C11	1.390 (2)
S1—O6	1.4568 (10)	C10—H10	0.9500
S1—O4	1.4631 (10)	C11—C12	1.3975 (18)
S1—C13	1.7702 (12)	C11—H11	0.9500
O2—H2A	0.86 (2)	C12—H12	0.9500
O2—H2B	0.85 (2)	C13—C18	1.3931 (17)
O3—H3A	0.84 (2)	C13—C14	1.3935 (17)
O3—H3B	0.79 (2)	C14—C15	1.3919 (18)
C1—C6	1.3819 (17)	C14—H14	0.9500
C1—C2	1.3888 (18)	C15—C16	1.3973 (18)
C2—C3	1.3923 (19)	C15—H15	0.9500
C2—H2	0.9500	C16—C17	1.3970 (18)
C3—C4	1.385 (2)	C16—C19	1.5037 (19)
C3—H3	0.9500	C17—C18	1.3888 (18)
C4—C5	1.385 (2)	C17—H17	0.9500
C4—H4	0.9500	C18—H18	0.9500
C5—C6	1.3942 (18)	C19—H19A	0.9800
C5—H5	0.9500	C19—H19B	0.9800
C6—H6	0.9500	C19—H19C	0.9800
			
O1—I1—C1	94.57 (4)	C1—C6—H6	121.3
O1—I1—C7	96.08 (5)	C5—C6—H6	121.3
C1—I1—C7	95.36 (4)	C8—C7—C12	123.12 (12)
O1—I1—O2	175.27 (4)	C8—C7—I1	114.46 (9)
C1—I1—O2	81.83 (4)	C12—C7—I1	122.41 (9)
C7—I1—O2	87.34 (4)	C7—C8—C9	118.05 (12)
O1—I1—O4	77.55 (4)	C7—C8—H8	121.0
C1—I1—O4	171.59 (4)	C9—C8—H8	121.0
C7—I1—O4	82.72 (4)	C10—C9—C8	120.41 (13)
O2—I1—O4	106.19 (3)	C10—C9—H9	119.8
O1—I1—O3	87.53 (4)	C8—C9—H9	119.8
C1—I1—O3	84.96 (4)	C9—C10—C11	120.17 (13)
C7—I1—O3	176.33 (4)	C9—C10—H10	119.9
O2—I1—O3	89.09 (3)	C11—C10—H10	119.9
O4—I1—O3	97.47 (3)	C10—C11—C12	120.71 (13)
O5—S1—O6	113.54 (7)	C10—C11—H11	119.6
O5—S1—O4	112.68 (7)	C12—C11—H11	119.6
O6—S1—O4	111.76 (6)	C7—C12—C11	117.53 (12)
O5—S1—C13	106.12 (6)	C7—C12—H12	121.2
O6—S1—C13	106.66 (6)	C11—C12—H12	121.2
O4—S1—C13	105.37 (6)	C18—C13—C14	120.40 (11)
I1—O2—H2A	112.9 (14)	C18—C13—S1	119.17 (9)
I1—O2—H2B	125.5 (14)	C14—C13—S1	120.33 (9)
H2A—O2—H2B	110 (2)	C15—C14—C13	119.13 (11)
I1—O3—H3A	124.2 (14)	C15—C14—H14	120.4
I1—O3—H3B	123.4 (15)	C13—C14—H14	120.4
H3A—O3—H3B	101.5 (19)	C14—C15—C16	121.38 (12)
S1—O4—I1	138.26 (6)	C14—C15—H15	119.3
C6—C1—C2	123.26 (11)	C16—C15—H15	119.3
C6—C1—I1	121.86 (9)	C17—C16—C15	118.41 (12)
C2—C1—I1	114.81 (9)	C17—C16—C19	120.65 (12)
C1—C2—C3	117.98 (13)	C15—C16—C19	120.93 (12)
C1—C2—H2	121.0	C18—C17—C16	120.94 (12)
C3—C2—H2	121.0	C18—C17—H17	119.5
C4—C3—C2	120.03 (13)	C16—C17—H17	119.5
C4—C3—H3	120.0	C17—C18—C13	119.73 (12)
C2—C3—H3	120.0	C17—C18—H18	120.1
C5—C4—C3	120.63 (12)	C13—C18—H18	120.1
C5—C4—H4	119.7	C16—C19—H19A	109.5
C3—C4—H4	119.7	C16—C19—H19B	109.5
C4—C5—C6	120.63 (13)	H19A—C19—H19B	109.5
C4—C5—H5	119.7	C16—C19—H19C	109.5
C6—C5—H5	119.7	H19A—C19—H19C	109.5
C1—C6—C5	117.46 (12)	H19B—C19—H19C	109.5
			
O5—S1—O4—I1	5.82 (11)	C10—C11—C12—C7	−0.16 (19)
O6—S1—O4—I1	135.08 (8)	O5—S1—C13—C18	−36.61 (12)
C13—S1—O4—I1	−109.46 (9)	O6—S1—C13—C18	−157.97 (10)
C6—C1—C2—C3	0.8 (2)	O4—S1—C13—C18	83.11 (11)
I1—C1—C2—C3	177.82 (11)	O5—S1—C13—C14	147.06 (11)
C1—C2—C3—C4	−0.7 (2)	O6—S1—C13—C14	25.70 (12)
C2—C3—C4—C5	0.3 (2)	O4—S1—C13—C14	−93.22 (11)
C3—C4—C5—C6	−0.1 (2)	C18—C13—C14—C15	−0.18 (19)
C2—C1—C6—C5	−0.6 (2)	S1—C13—C14—C15	176.11 (10)
I1—C1—C6—C5	−177.39 (10)	C13—C14—C15—C16	−0.6 (2)
C4—C5—C6—C1	0.2 (2)	C14—C15—C16—C17	1.0 (2)
C12—C7—C8—C9	−0.70 (19)	C14—C15—C16—C19	−177.83 (13)
I1—C7—C8—C9	−179.34 (10)	C15—C16—C17—C18	−0.7 (2)
C7—C8—C9—C10	0.4 (2)	C19—C16—C17—C18	178.17 (13)
C8—C9—C10—C11	−0.1 (2)	C16—C17—C18—C13	−0.1 (2)
C9—C10—C11—C12	−0.1 (2)	C14—C13—C18—C17	0.51 (19)
C8—C7—C12—C11	0.56 (19)	S1—C13—C18—C17	−175.82 (10)
I1—C7—C12—C11	179.10 (9)		

### Hydrogen-bond geometry (Å, º)

**Table:**

*D*—H···*A*	*D*—H	H···*A*	*D*···*A*	*D*—H···*A*
O2—H2*A*···O3^i^	0.86 (2)	1.92 (2)	2.7563 (14)	165 (2)
O2—H2*B*···I1^ii^	0.85 (2)	3.14 (2)	3.9397 (10)	158.1 (18)
O2—H2*B*···O1^ii^	0.85 (2)	1.91 (2)	2.7443 (14)	169 (2)
O3—H3*A*···S1^i^	0.84 (2)	3.00 (2)	3.7847 (11)	155.7 (17)
O3—H3*A*···O5^i^	0.84 (2)	1.92 (2)	2.7625 (15)	174.6 (19)
O3—H3*B*···S1^iii^	0.79 (2)	2.89 (2)	3.5812 (10)	147.5 (18)
O3—H3*B*···O6^iii^	0.79 (2)	1.99 (2)	2.7776 (14)	171 (2)
C2—H2···O1	0.95	2.49	2.9805 (16)	112
C6—H6···O2	0.95	2.33	2.9408 (17)	122
C6—H6···O6^i^	0.95	2.58	3.2562 (16)	129
C8—H8···O1	0.95	2.38	2.9521 (17)	119
C12—H12···O2	0.95	2.41	3.0957 (17)	129
C14—H14···O5^iv^	0.95	2.65	3.4301 (16)	139

Symmetry codes: (i) −*x*+1, −*y*+1, −*z*+1; (ii) *x*−1, *y*, *z*; (iii) −*x*+2, −*y*+1, −*z*+1; (iv) *x*+1, *y*, *z*.

## References

[bb1] Bondi, A. (1964). *J. Phys. Chem.* **68**, 441–451.

[bb2] Bozopoulos, A. P. & Rentzeperis, P. J. (1987). *Acta Cryst.* C**43**, 142–144.

[bb3] Bruker (2021). *APEX4*, *SADABS* and *SAINT*, Bruker Nano Inc., Madison, Wisconsin, USA.

[bb4] Chen, Y. (2007). PhD thesis, The University of Akron, Akron, Ohio, United States.

[bb5] Koser, G. F., Wettach, R. H., Troup, J. M. & Frenz, B. A. (1976). *J. Org. Chem.* **41**, 3609–3611.

[bb6] Macrae, C. F., Sovago, I., Cottrell, S. J., Galek, P. T. A., McCabe, P., Pidcock, E., Platings, M., Shields, G. P., Stevens, J. S., Towler, M. & Wood, P. A. (2020). *J. Appl. Cryst.* **53**, 226–235. 10.1107/S1600576719014092 PMC6998782 32047413

[bb7] Schröckeneder, A., Stichnoth, D., Mayer, P. & Trauner, D. (2012). *Beilstein J. Org. Chem.* **8**, 1523–1527. 10.3762/bjoc.8.172 PMC3458777 23019487

[bb8] Sheldrick, G. M. (2015*a*). *Acta Cryst.* A**71**, 3–8.

[bb9] Sheldrick, G. M. (2015*b*). *Acta Cryst.* C**71**, 3–8.

[bb10] Westrip, S. P. (2010). *J. Appl. Cryst.* **43**, 920–925.

[bb11] Yusubov, M. S., Svitich, D. Y., Yoshimura, A., Nemykin, V. N. & Zhdankin, V. V. (2013). *Chem. Commun.* **49**, 11269–11271. 10.1039/c3cc47090c 24153437

[bb12] Zhdankin, V. V., Erickson, S. A. & Hanson, K. J. (1997). *J. Am. Chem. Soc.* **119**, 4775–4776.

